# Full automation of total metabolic tumor volume from FDG-PET/CT in DLBCL for baseline risk assessments

**DOI:** 10.1186/s40644-022-00476-0

**Published:** 2022-08-12

**Authors:** S. Jemaa, J. N. Paulson, M. Hutchings, L. Kostakoglu, J. Trotman, S. Tracy, A. de Crespigny, R. A. D. Carano, T. C. El-Galaly, T. G. Nielsen, T. Bengtsson

**Affiliations:** 1 1PHC Imaging, Genentech, Inc, South San Francisco, CA USA; 2 Biostatistics, Genentech, Inc, South San Francisco, CA USA; 3 Department of HaematologyRigshospitalet, Copenhagen, Denmark; 4 Department of Radiology and Medical Imaging, University of Virginia, Charlottesville, VA USA; 5 Department of Haematology, Concord Repatriation General Hospital, University of Sydney, Concord, NSW Australia; 6 Clinical Imaging Group, Genentech, Inc, South San Francisco, CA USA; 7 Department of Hematology, Aalborg University Hospital, Aalborg, Denmark; 8 Pharmaceutical Development Clinical Oncology, F. Hoffmann-La Roche Ltd, Bldg 1, Grenzarcherstrasse 124m, CH-4070 Basel, Switzerland; 9 Department of Statistics, University of California-Berkeley, Berkeley, CA USA

**Keywords:** DLBCL, FDG-PET, Imaging, AI

## Abstract

**Background:**

Current radiological assessments of ^18^fluorodeoxyglucose-positron emission tomography (FDG-PET) imaging data in diffuse large B-cell lymphoma (DLBCL) can be time consuming, do not yield real-time information regarding disease burden and organ involvement, and hinder the use of FDG-PET to potentially limit the reliance on invasive procedures (e.g. bone marrow biopsy) for risk assessment.

**Methods:**

Our aim is to enable real-time assessment of imaging-based risk factors at a large scale and we propose a fully automatic artificial intelligence (AI)-based tool to rapidly extract FDG-PET imaging metrics in DLBCL. On availability of a scan, in combination with clinical data, our approach generates clinically informative risk scores with minimal resource requirements. Overall, 1268 patients with previously untreated DLBCL from the phase III GOYA trial (NCT01287741) were included in the analysis (training: *n* = 846; hold-out: *n* = 422).

**Results:**

Our AI-based model comprising imaging and clinical variables yielded a tangible prognostic improvement compared to clinical models without imaging metrics. We observed a risk increase for progression-free survival (PFS) with hazard ratios [HR] of 1.87 (95% CI: 1.31–2.67) vs 1.38 (95% CI: 0.98–1.96) (C-index: 0.59 vs 0.55), and a risk increase for overall survival (OS) (HR: 2.16 (95% CI: 1.37–3.40) vs 1.40 (95% CI: 0.90–2.17); C-index: 0.59 vs 0.55). The combined model defined a high-risk population with 35% and 42% increased odds of a 4-year PFS and OS event, respectively, versus the International Prognostic Index components alone. The method also identified a subpopulation with a 2-year Central Nervous System (CNS)-relapse probability of 17.1%.

**Conclusion:**

Our tool enables an enhanced risk stratification compared with IPI, and the results indicate that imaging can be used to improve the prediction of central nervous system relapse in DLBCL. These findings support integration of clinically informative AI-generated imaging metrics into clinical workflows to improve identification of high-risk DLBCL patients.

**Trial Registration:**

Registered clinicaltrials.gov number: NCT01287741.

**Graphical Abstract:**

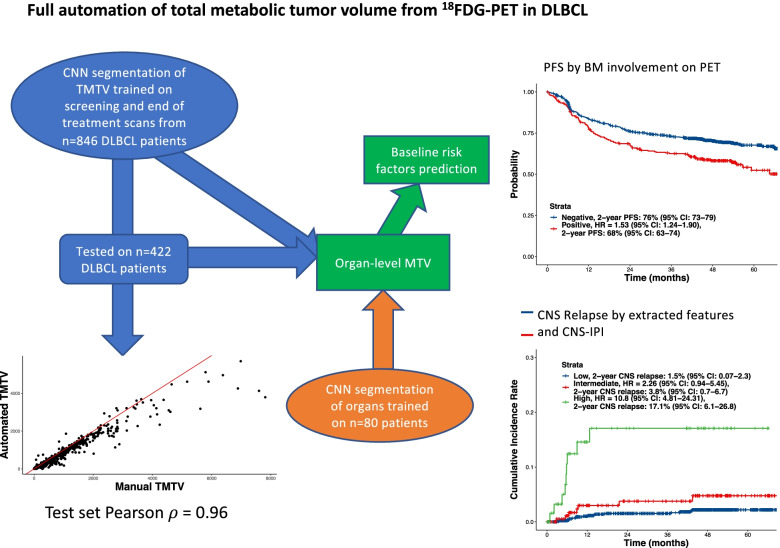

**Supplementary Information:**

The online version contains supplementary material available at 10.1186/s40644-022-00476-0.

## Introduction

Diffuse large B-cell lymphoma (DLBCL) is the most common subtype of non-Hodgkin lymphoma (NHL), accounting for 30–40% of all cases [1]. Current first-line standard of care consists of rituximab, cyclophosphamide, doxorubicin, vincristine, and prednisone (R-CHOP) combination therapy; however, patient outcomes vary due to the genotypic and phenotypic heterogeneity of DLBCL [2, 3]. While R-CHOP induces durable responses in up to 70% of patients, the identification of the sizeable minority of patients with insufficient response to R-CHOP is a clinical challenge [4]. Patients with relapsed/refractory DLBCL have poor outcomes, with a previous study demonstrating a 1-year overall survival (OS) of only 41.6% in patients undergoing autologous stem cell transplantation [5]. Patients with primary refractory disease generally have the worst prognosis, with only 20% surviving for more than 2 years [6]. However, with chimeric antigen receptor T-cell therapy patients refractory to chemotherapy have shown high response rate and overall survival in the second line setting for transplant candidates (2-year OS of 61%) [7] and for patients not eligible for transplant or later relapse (1 year OS of 49%) [8].

Current clinical prognostic risk-stratification models are not optimal for the identification of patients with the highest risk for treatment failure, who should be considered for alternative treatment strategies, including clinical trials with experimental therapies. The widely used International Prognostic Index (IPI) is based on patient parameters, including performance status, age, Ann Arbor stage, number of extranodal sites, and serum lactate dehydrogenase (LDH) levels [9]. This model gives suboptimal sensitivity and specificity in identifying patients who are unlikely to achieve durable remission; for example, a previous study demonstrated that patients with a 3-year progression-free survival (PFS) or OS below 50% were unable to be identified by IPI [10]. Variations of the IPI, such as the National Comprehensive Cancer Network (NCCN)-IPI, have been developed with limited improvement in prognostic performance [11].

 [12]Fluorodeoxyglucose-positron emission tomography (FDG-PET) is a commonly used imaging tool in oncology, where metabolic activity of tumors can be assessed by three-dimensional measurements of the tracer uptake distribution [13–15]. While FDG uptake can vary considerably across different lymphoma types, aggressive NHLs with fast tumor growth generally exhibit high FDG uptake, making PET/computed tomography (CT) particularly powerful for the visualization of these subtypes [13, 16]. The distribution and volume of disease on FDG-PET/CT imaging have been shown to be prognostic of clinical outcome in DLBCL [17], with high tumor volumes associated with poorer outcomes [18]. The recently proposed International Metabolic Prognostic Index (IMPI) shows that manually assessed TMTV adds sensitivity to IPI for high risk patient identification in DLBCL [19].

The determination of total metabolic tumor volume (TMTV) is generally based on semi-automatically defined volumes of interest (VOI) around the tumor using a software visualization program; however, often these VOIs are manually adjusted by the nuclear medicine specialist to include the entire tumor and avoid healthy tissues. Overall, this process is time-consuming, labor intensive and observer-dependent. The determination of other imaging metrics, e.g. bone marrow involvement or extra-nodal involvement, may be individually faster to extract manually but time limitations do not allow the extraction of a large set of such metrics. Leveraging deep learning for automated disease identification on FDG-PET/CT images enables global and location-/organ-specific measurements of disease burden in NHL, and provides fast collection of highly granular, patient-level (metabolically active) tumor information. Tumor characterization assessed by fast, transparent, and reproducible automated algorithms [20–, 21–23] could add important prognostic information to currently used clinical risk scores and improve the identification of high-risk patients who are unlikely to respond to standard therapy. It is therefore desirable to streamline the evaluation of imaging-based risk factors to allow for the evaluation of imaging evidence in the care of DLBCL at a scale that was previously not possible.

Statistical models that combine clinical and imaging markers may improve prognostication in DLBCL, thus enabling risk-adapted treatment strategies at the time of diagnosis. These models may also support faster development of novel treatment strategies that are more effective in patients at high risk of progression or relapse. The primary objective of this study was to evaluate baseline automated FDG-PET/CT imaging metrics in combination with clinical risk factors in modeling prognosis for patients with de novo DLBCL. Secondary objectives included prediction of central nervous system (CNS) relapse, and an evaluation of whether a fully automated algorithm could reproduce the prognostic relevance of bone marrow and other extranodal involvement in DLBCL.

## Methods

### Patients

This study analyzed data from the randomized phase III GOYA trial (NCT01287741), a multicenter, open-label trial comparing the efficacy of obinutuzumab (G; GA101) in combination with CHOP (G-CHOP) versus R-CHOP in 1418 previously untreated patients with CD20-positive DLBCL [24]. The GOYA trial identified no significant difference in survival outcomes between the G-CHOP and R-CHOP treatment arms [24, 25], and so the arms were combined for the present analysis. The study design and eligibility criteria for the GOYA trial have been previously described [25].

The trial was conducted in accordance with the Declaration of Helsinki and the International Conference on Harmonization of Good Clinical Practice guidelines. Trial protocol approval was obtained from the ethics committee/institutional review board at each participating institution and written informed consent to participate was provided by all patients.

### FDG-PET/CT imaging

Baseline FDG-PET/CT imaging was performed 1–35 days prior to the first dose of study treatment (R-CHOP or G-CHOP) at study sites where a PET/CT scanner was available [24]. All PET/CT scans were performed according to a standardized imaging protocol, centrally collected and segmented using a semi-automated workflow (MIM Software Inc, OH, US) by an independent review facility, as previously described [12]. Three experienced nuclear medicine physicians reviewed baseline PET/CT scans collected in real time during the study. TMTV was calculated using a tumor threshold of 1.5 times the mean SUV of the liver + 2 standard deviations. Only those tumors that measured > 1 mL were included in the TMTV calculation. FDG-PET/CT images were analyzed using a deep learning algorithm, as developed and defined by Jemaa et al. (performed at Genentech, Inc.) [20]. The method does not require the user to indicate a specific intensity threshold for each scan. The models are trained to recognize tumors on both spatial and intensity information in the 1.5*liver SUV + 2*standard deviations TMTV thresholded ground truth segmentation by radiologists. Total and by-organ/-location number and volume of lesions were extracted using the generated tumor and organ/-location masks. The models in this analysis were trained on the training set summarized below including 624 scans without metabolically active lesions. Tumor segmentation and organ and location segmentation are described in the Supplementary Methods and Supplemental Table 1.

### Endpoints and assessments

We assessed and quantified the association between baseline automated imaging metrics and clinical outcome, including investigator-assessed PFS and OS, and determined whether the addition of imaging metrics to clinical variables can improve outcome risk prediction and in particular identification of patients at high risk at baseline.

We further evaluated whether a fully automated algorithm could reproduce the prognostic relevance of extranodal and bone marrow involvement observed using visual or PET/CT assessments [26, 27]. The correlation of extranodal disease and automated TMTV (aTMTV) was also explored to determine whether high extranodal disease burden was strongly correlated to high overall aTMTV. Additionally, the association of extranodal disease with outcome was examined through the comparison of outcomes in patients by the number of extranodal sites involved (by aTMTV). The association between automatically extracted imaging information and CNS relapse, using survival analysis and data from Klanova et al [28] was evaluated. The correlation between aTMTV and other clinically relevant measures (LDH and Ann Arbor stage) was investigated as an exploratory endpoint, as well as the correlation between LDH and extranodal involvement.

### Statistical analysis

A training and hold-out set were developed for reproducibility and prognostic modeling activities for GOYA. The general strategy in defining a hold-out set is to select a prespecified proportion (33%) of trial participants and balance for important study-specific variables. The balancing variables used for the GOYA trial included the antibody received (G or R), IPI risk category (high, intermediate-high, or low), number of PFS events, number of OS events, and manual TMTV (quartiles and not available).

Tumor segmentation CNNs were trained on screening and end of treatment scans while statistical analyses were applied only for screening scans.

As a practical approach, imaging metrics were dichotomized as to location involvement/no-involvement, by extranodal sites > 1 (as done in IPI), or by quartile splits (i.e.,, TMTV, number of total lesions.) For LDH and hemoglobin we have used the ULN and LLN respectively, while 65 years age is considered the threshold for elderly by the FDA. In addition, as extensively used risk factors in DLBCL, we also explore IPI > 2 and ECOG PS > 1 in our analysis. The selection of the best prognostic models was done as follows. A univariate `sweep’ was first performed in the training set by retaining statistically significant univariate factors. For joint variable selection, again using the training set, the univariately significant variables were then submitted to a multivariate LASSO Cox PH regression [29], with the best performing model identified using fivefold cross-validation. The variables identified by the preceding LASSO step were used to fit a multivariate Cox PH model on the training set, i.e. the ‘final model’’. This model was finally applied out-of-sample on the test set. Model performance was evaluated by area under the receiver operating characteristic curve (AUC) and C-index on the hold-out population. For extranodal involvement, the algorithm estimated the mass of the lung, liver, spleen, and bone lymphoma involvement, which are among the more commonly involved extranodal areas in DLBCL [10]. The joint multivariate Cox PH models were used to calculate hazard ratios (HRs) and the 95% confidence intervals (95% CIs) to compare model performance. From the training set, the cut-off for the risk score was defined as the median risk score. Kaplan–Meier estimates were used to analyze time-to-event data. All statistical analyses and tests were performed using R 3.6.3 and the Survival package (the Wald test was used for the univariate and multivariate models and log rank tests were used for the survival curves) [30].

## Results

### Patient demographics and disease characteristics

Overall, 1418 patients were enrolled in the GOYA trial and 1407 were included in the safety-evaluable population (SEP); of these, 1268 patients with baseline FDG-PET/CT images available for automated assessment of imaging metrics were included in the evaluable population (EP). Patients excluded from the EP included 99 patients without screening FDG-PET/CT scans, 15 patients with no Attenuation Corrected CT scans and 25 patients whose FDG-PET scans could not be processed. Patients were further divided into predefined training (*n* = 846) and hold-out populations (*n* = 422). Patient demographics and clinical characteristics were similar between the GOYA SEP, the EP, and the non-EP (Table 1). No clinically relevant difference in outcome was observed for patients in the SEP versus patients in the EP; however, a higher survival probability was observed for the EP compared with the non-EP (Supplemental Fig. 1). The median duration of follow-up for all patients was 48.2 months (range, 0.16–78.2).

**Table 1:** Patient demographics and clinical characteristics for the SEP (GOYA), the EP and the non-EP

	**Safety-evaluable (GOYA)** **(*N* = 1407)**	**EP** **(*N* = 1268)**	**Non-EP** **(*N* = 139)**
**Treatment, n (%)**			
G-CHOP	704 (50)	636 (50)	68 (49)
R-CHOP	703 (50)	632 (50)	71 (51)
**Age, years**			
Mean	59	59	58
Median	62	62	60
Min–max	18–86	18–86	19–81
**Sex, n (%)**			
Female	663 (47)	595 (47)	68 (49)
Male	744 (53)	673 (53)	71 (51)
**IPI at baseline, n (%)**			
0–2	781 (56)	709 (56)	72 (52)
3–5	626 (44)	559 (44)	67 (48)
**ECOG PS, n (%)**			
0–1	1222 (87)	1110 (88)	112 (81)
≥ 2	184 (13)	157 (12)	27 (19)
Missing	1 (0)	1 (0)	0
**Serum LDH at baseline, n (%)**			
Elevated	811 (58)	724 (57)	87 (63)
Normal	592 (42)	540 (43)	52 (37)
Missing	4 (0)	4 (0)	0
**Hemoglobin, n (%)**			
< LLN	702 (50)	627 (49)	64 (46)
≥ LLN	705 (50)	641 (51)	75 (54)
**Ann Arbor stage, n (%)**			
I–II	339 (24)	302 (24)	37 (27)
III–IV	1068 (76)	966 (76)	102 (73)
**Extranodal involvement, n (%)**			
≤ 1	910 (65)	815 (64)	95 (68)
> 1	497 (35)	453 (36)	44 (32)

*ECOG PS* Eastern Cooperative Oncology Group performance status, *EP* Evaluable population, *G-CHOP* Obinutuzumab plus cyclophosphamide, doxorubicin, vincristine and prednisone; obinutuzumab, *IPI* International Prognostic Index, *LDH* lactate dehydrogenase, *LLN* lower limit of normal, *R-CHOP* Rituximab plus cyclophosphamide, doxorubicin, vincristine and prednisone, *SEP* Safety-evaluable population

**Fig. 1:**
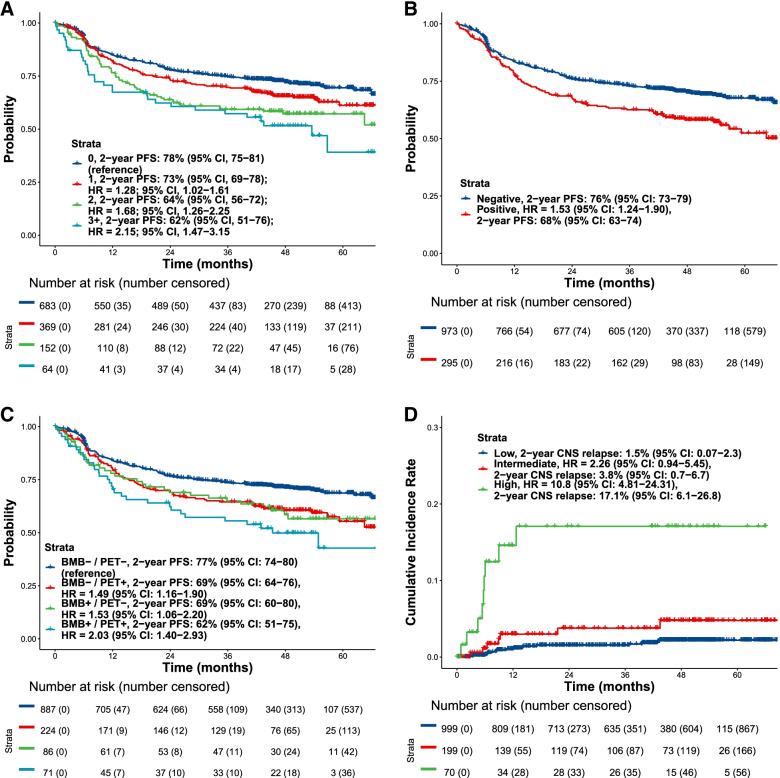
PFS in the full EP by (**A**) number of extranodal sites*, (**B**) bone involvement by PET/CT and (**C**) baseline bone marrow biopsy and bone involvement by PET/CT, and (**D**) CNS relapse by the model incorporating imaging metrics and CNS-IPI. *Extranodal involvement was estimated by combining tumor masses in the lungs, liver, spleen, and bone tumor involvement. BMB, bone marrow biopsy; CI, confidence interval; CNS, central nervous system; CNS-IPI, CNS International Prognostic Index; CT, computed tomography; EP, evaluable population; IPI, International Prognostic Index; HR, hazard ratio; PET, positron-emission tomography; PFS, progression-free survival

aTMTV (Median = 279 mL, range 0-9693 mL) correlated highly with manual TMTV (Median = 347 mL, range 0-9694 mL; Spearman’s ρ = 0.96), the DICE score on the test set was 0.88 and the average number of true and false positive lesions detected per scan were 7.08 and 0.61, respectively, in the test set. The average number of false negative lesions per scan was 0.82 in the test set. An increased number of extranodal sites correlated with high aTMTV (Supplemental Fig. 2). An evaluation of the association between the extent of extranodal involvement and PFS showed that the risk of disease progression was greater with increasing numbers of extranodal sites (Supplemental Table 2; Fig. 1A). Similar results were observed in a sensitivity analysis considering splenic lesions as nodal for both the association between extranodal involvement and aTMTV, and extranodal involvement and PFS.

aTMTV trended towards an association with LDH (Spearman *ρ* = 0.503, Supplemental Fig. 3A; a correlation between LDH and extranodal involvement was also observed [Supplemental Fig. 3B]) as was a correlation between Ann Arbor stage and aTMTV (Supplemental Fig. 4).

Positive bone marrow involvement observed with FDG-PET/CT, was also associated with reduced PFS, with patients testing positive by fully automated assessment being at a higher risk of disease progression (2-year PFS probability, 68%; 95% CI, 63–74) than those who were negative (2-year PFS probability, 76%; 95% CI, 73–79; *P* < 0.0001; Fig. 1B). Similarly, a positive baseline bone marrow biopsy was a risk factor for inferior PFS, particularly when accompanied by positive bone marrow involvement on PET/CT (Fig. 1C; Supplemental Table 3).

### Prognostic model comprising imaging parameters combined with clinical variables for predictability of CNS relapse and disease outcome

**Fig. 2:**
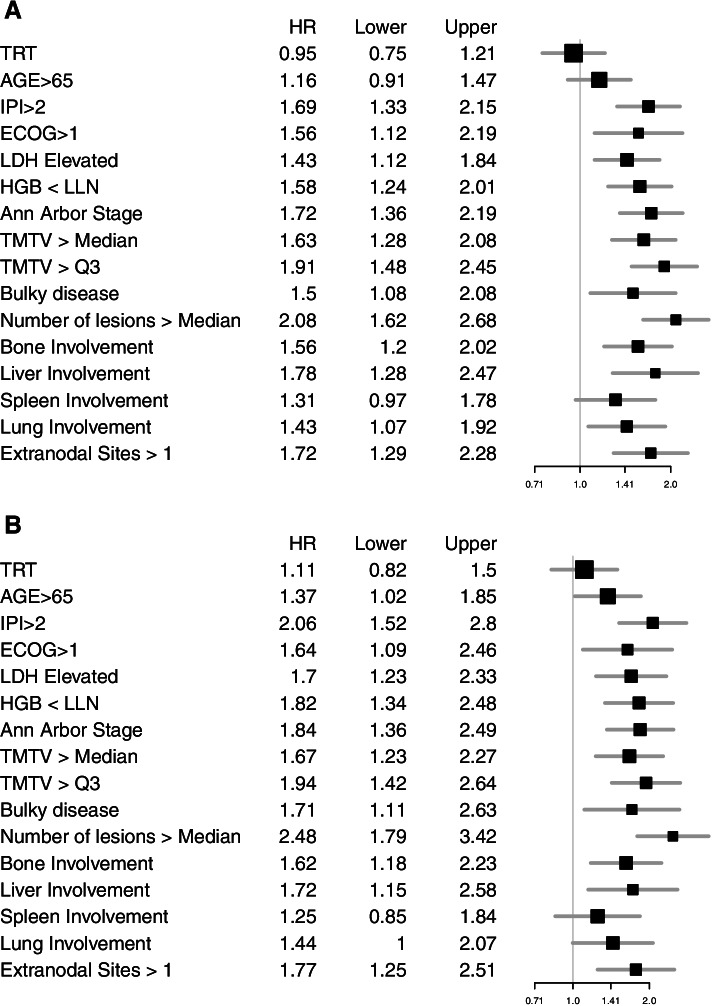
Forest plots for univariate HRs with 95% CIs for (**A**) investigator-assessed PFS and (**B**) OS in the training set (*n* = 846). Involvement was defined as the presence of at least one FDG-avid lesion in the organ or location of interest. CI, confidence interval; ECOG PS, Eastern Cooperative Oncology Group performance status; FDG, fluorodeoxyglucose; G-CHOP, obinutuzumab plus cyclophosphamide, doxorubicin, vincristine, and prednisone; HR, hazard ratio; IPI, International Prognostic Index; LDH, lactate dehydrogenase; LLN, lower limit of normal; OS, overall survival; PFS, progression-free survival; Q3, third quartile; TMTV, total metabolic tumor volume

The results for the univariate variable screen for PFS (Fig. 2) indicated that all variables except treatment, spleen involvement and age > 65 years were statistically significant (*P* < 0.05). For PFS, the LASSO Cox regression identified a model comprising three imaging metrics (aTMTV > third quartile, number of lesions > 4 [median], and liver involvement) combined with four standard clinical variables (hemoglobin < lower limit of normal, Eastern Cooperative Oncology Group Performance Status (ECOG PS) > 1, Ann Arbor stage IV, LDH > upper limit of normal; Supplemental Fig. 5). HRs and CIs for the imaging metrics and clinical variables in the identified multivariate Cox PH model and the 4-year PFS probability are listed in Table 2. The model showed improved performance (AUC = 0.61, C-index = 0.63 [hold-out set]) compared with the model comprising standard clinical variables only (AUC = 0.57, C-index = 0.59 [hold-out set]; Supplemental Fig. 6). Of note, in the multivariate model, Ann Arbor stage IV (HR, 1.49; 95% CI, 1.08–1.96; *P* = 0.01) and number of lesions > 4 (median; HR, 1.91; 95% CI, 1.34–2.71; *P* < 0.001) were identified as independent parameters of significant value for predicting PFS.

**Table 2:** HRs and CIs for PFS for the multivariate Cox PH model with imaging metrics and the multivariate Cox PH model with clinical variables only (training set)

	**PFS**					**OS**				
	**HR**	**95% CI**	***P*-value**	**C-Index** **test set**	**AUC 2-years** **test set**	**HR**	**95% CI**	***P*-value**	**C-Index test set**	**AUC 2-years test set**
**Cox PH model with imaging metrics**										
Hemoglobin < LLN	1.22	0.94–1.58	.13			1.37	0.99–1.89	.06		
ECOG PS > 1	1.26	0.89–1.78	.19			1.29	0.84–1.96	.24		
Ann Arbor stage IV	1.41	1.10–1.82	.007			1.49	1.08–2.04	.01		
aTMTV > Q3	1.31	0.98–1.74	.06	0.63	0.59	1.18	0.83–1.67	.35	0.62	0.61
Number of lesions > 4 (median)	1.62	1.23–2.71	.0006			1.91	1.34–2.71	.0003		
Liver involvement	1.16	0.82–1.64	.40			1.03	0.68–1.58	.88		
LDH elevated	1.12	0.86–1.46	.39			1.30	0.93–1.82	.12		
**Cox PH model with clinical variables only**										
Hemoglobin < LLN	1.40	1.09–1.80	.01			1.57	1.14–2.15	.005		
ECOG PS > 1	1.40	0.99–1.96	.05	0.59	0.57	1.39	0.92–2.10	.11	0.59	0.57
Ann Arbor stage IV	1.61	1.26–2.05	.0001			1.68	1.24–2.28	.0009		
LDH elevated	1.30	1.01–1.68	.04			1.51	1.09–2.09	.01		

*CI* confidence interval, *ECOG PS* Eastern Cooperative Oncology Group performance status, *HR* Hazard ratio, *LDH* Lactate dehydrogenase, *LLN* Lower limit of normal, *OS* Overall survival, *PFS* Progression-free survival, *PH* Proportional hazard, *Q3* Third quartile, *TMTV* Total metabolic tumor volume

The high-risk population from the multivariate prognostic model that combined imaging and clinical variables was associated with a significantly increased risk of CNS relapse compared with the low-risk population (by median split; HR, 5.42; 95% CI, 2.24–13.14). The 2-year CNS-relapse probability for the high-risk population was 5.0% (95% CI, 3.0–6.9), and 0.5% (95% CI, 0.0–1.1) for the low-risk population. We combined the CNS International Prognostic Index (CNS-IPI) and the model incorporating imaging and clinical variables to define three levels of risk. The high-risk population was defined as patients with a high-risk score by CNS-IPI and a risk score from the model incorporating imaging variables above the 90^th^ percentile. This combined model identified a subpopulation with a 2-year CNS-relapse probability of 17.1% (95% CI, 6.1–26.8; Fig. 1D).

**Fig. 3:**
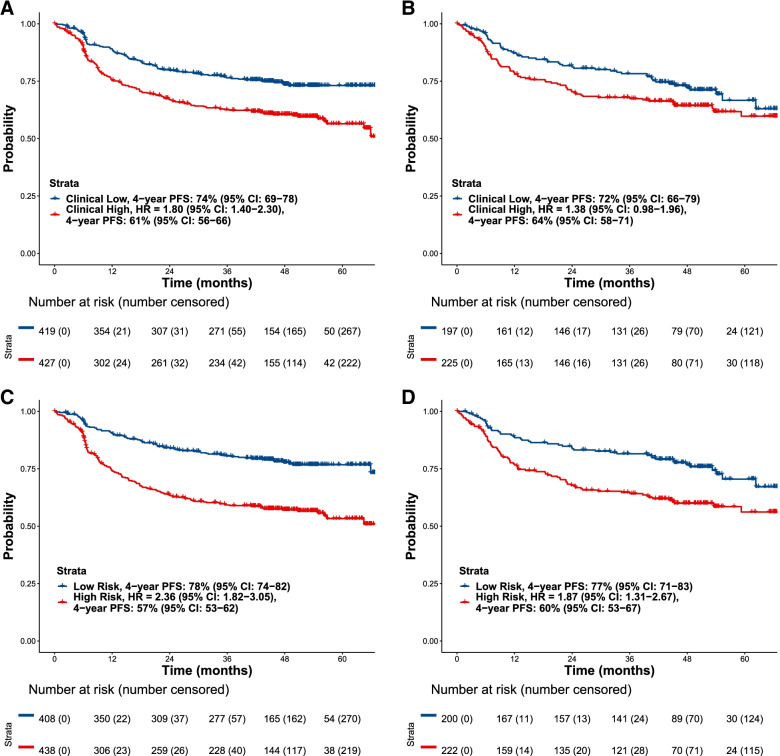
Investigator-assessed PFS by clinical variables in (**A**) the training (*N* = 846) and (**B**) the hold-out set (*N* = 422), and (**C**) the model incorporating imaging metrics in the training and (**D**) the hold-out set. CI, confidence interval; HR, hazard ratio; PFS, progression-free survival

In both the training and the hold-out set, patients at high risk in both the model comprising clinical parameters alone (Figs. 3A and B, respectively) and the model including imaging metrics (Figs. 3C and D, respectively) had a worse PFS compared with patients at low risk. In the hold-out set, the model identified patients with an increased PFS risk (HR, 1.87; 95% CI, 1.31–2.67; Fig. 3D) compared with the model with clinical variables alone (HR, 1.38; 95% CI, 0.98–1.96; Fig. 3B), and an increased OS risk (HR, 2.16; 95% CI, 1.37–3.40) compared with clinical variables alone (HR, 1.40; 95% CI, 0.90–2.17; Table 3).

**Table 3:** HRs and 2-, 3- and 4-year PFS and OS probabilities for patients at high and low risk in the hold-out set for the model with imaging and clinical variables and the model with clinical variables only

	**PFS**					**OS**				
	***N***	**HR (95% CI)**	**2-year PFS probability,** **% (95% CI)**	**3-year PFS probability,** **% (95% CI)**	**4-year PFS probability,** **% (95% CI)**	***n***	**HR (95% CI)**	**2-year OS probability,** **% (95% CI)**	**3-year OS probability,** **% (95% CI)**	**4-year OS probability,** **% (95% CI)**
**Model with imaging and clinical variables**										
Low-risk patients	200	*Reference*	84 (79–90)	82 (76–87)	77 (71–83)	210	*Reference*	90 (86–94)	89 (85–94)	88 (84–93)
High-risk patients	222	1.87 (1.31–2.67)	68 (62–74)	65 (58–72)	60 (53–67)	212	2.16 (1.37–3.40)	79 (74–85)	76 (70–82)	75 (69–81)
**Model with clinical variables only**										
Low-risk patients	197	*Reference*	82 (76–87)	78 (73–84)	72 (66–79)	197	*Reference*	88 (83–92)	86 (81–91)	85 (80–90)
High-risk patients	225	1.38 (0.98–1.96)	70 (64–77)	68 (62–75)	64 (58–71)	225	1.40 (0.90–2.17)	82 (77–87)	80 (75–85)	78 (73–84)

*CI* Confidence interval, *HR* Hazard ratio, *OS* Overall survival, *PFS* Progression-free survival

In the hold-out set, the model incorporating imaging metrics defined a high-risk population with a 35% and 42% increase in odds of 4-year PFS and 4-year OS events, respectively, compared to the model composed of the IPI components alone (age, ECOG PS, Ann Arbor stage, LDH elevated and number of extranodal sites). In terms of absolute risk, the high-risk group had a 4-year PFS probability of 60% (95% CI, 53–67) and the low-risk group had a 4-year PFS probability of 77% (95% CI, 71–83) in the model incorporating imaging metrics; while for the model with clinical parameters only, the probabilities for 4-year PFS were 64% (95% CI, 58–71) and 72% (95% CI, 66–79), respectively (Table 3).

The 4-year probability for OS for the model including the automatically extracted tumor parameters in the hold-out set was 75% (95% CI, 69–81) for the high-risk group and 88% (95% CI, 84–93) for the low-risk group (Table 3). For the model with clinical variables only, these OS probabilities were 78% (95% CI, 73–84) and 85% (95% CI, 80–90), respectively (Table 3). The results for the training set are reported in Table 4.

**Table 4:** HRs and 2-, 3- and 4-year PFS and OS probabilities for patients at high and low risk in the training set for the model with imaging and clinical variables and the model with clinical variables only

	**PFS**					**OS**				
	***N***	**HR (95% CI)**	**2-year PFS probability, % (95% CI)**	**3-year PFS probability,** **% (95% CI)**	**4-year PFS probability,** **% (95% CI)**	***n***	**HR (95% CI)**	**2-year OS probability,** **% (95% CI)**	**3-year OS probability,** **% (95% CI)**	**4-year OS probability,** **% (95% CI)**
**Model with imaging and clinical variables**										
Low-risk patients	408	*Reference*	84 (80–88)	81 (77–85)	78 (74–82)	422	*Reference*	91 (88–94)	90 (87–93)	89 (85–92)
High-risk patients	438	2.36 (1.82–3.05)	64 (59–68)	59 (55–64)	57 (53–62)	424	2.69 (1.93–3.73)	79 (75–83)	74 (69–78)	71 (67–76)
**Model with clinical variables only**										
Low-risk patients	419	*Reference*	80 (76–84)	76 (72–81)	74 (69–78)	419	*Reference*	90 (87–93)	88 (85–91)	86 (82–89)
High-risk patients	427	1.80 (1.40–2.30)	67 (63–72)	63 (58–68)	61 (56–66)	427	2.03 (1.48–2.78)	80 (76–84)	75 (71–80)	74 (70–78)

*CI* Confidence interval, *HR* Hazard ratio, *OS* Overall survival, *PFS* Progression-free survival

## Discussion

The availability of accurate prognostication tools for DLBCL is limited and, with the advent of various new treatment options and novel therapies, there is an unmet need for reliable methods to predict disease outcomes. The current study evaluated automated FDG-PET/CT imaging metrics, derived using artificial intelligence (AI), as potential granular-level, fast, and scalable tools for risk stratification. This method produces risk assessments in real-time upon scan availability. We analyzed whether incorporating these automated imaging parameters, alongside known prognostic clinical variables, for modeling PFS/OS in patients with de novo DLBCL could provide additional prognostic value in predicting disease outcome (OS/PFS). We showed that such metrics can provide a tangible improvement in risk stratification relative to standard clinical variables.

To assess the performance of our model comprising imaging and clinical variables, we compared it with IPI, which is currently a widely used tool in clinical practice and was applied as a stratification factor in the GOYA trial. We evaluated differences between PFS by IPI risk group and the risk score produced by the imaging/clinical metric-based model for the same subpopulation sizes as the IPI 3–5 subgroup (44%; Table 1). A greater difference between risk groups was observed for 2-year PFS and OS according to high or low risk for the model that included imaging metrics (17.5% for PFS and 7.4% for OS) compared with IPI risk groups 0–2 versus 3–5 (11.2% and 5.3%, respectively; data not shown). We note that the risk assessments produced by our model are useful only to identify faster-progressing patients who, per the training data, are more likely to be R/G-CHOP-resistant or refractory. Additional patient-level information, such as a patient’s gene expression or mutational profile, or tissue diagnostics, may enable matching individual patients to the best therapy, e.g. an appropriate targeted therapy.

The model incorporating automatically derived imaging metrics defined a new high-risk population with a 35% increase in the odds of a 4-year PFS event, compared with the population defined by the IPI components alone (hold-out set). An increased risk for PFS and for OS was also observed when imaging parameters were incorporated, compared with clinical parameters alone (hold-out set; PFS HR, 1.87 vs 1.38; OS HR, 2.16 vs 1.40, respectively).

Based on the results, assuming an annual rate of 18 000 newly diagnosed patients [31] with DLBCL in the US, the proposed model could potentially identify an additional 1350 patients at high risk, compared with IPI risk stratification alone. This result represents a tangible increase in model performance for a disease where current clinical risk models are suboptimal for identifying patients at high risk of early R-CHOP failure, which is associated with very poor outcomes [6]. In fact, clinical risk models such as IPI have stronger performance when identifying patients at low risk of disease progression [10]. These findings support the integration of additional information such as imaging metrics into the currently used clinical risk models to improve the identification of patients with de novo DLBCL at high risk. We note that having a fully automated model allows the addition of the imaging metrics delineated here in an efficient and fast manner.

We also assessed the recently published IMPI method to identify high risk patients in Goya. When comparing to IPI, based on the same subgroups sizes, this analysis showed that patients at high risk by IMPI had 3-years PFS and OS probabilities of 55% (48–62) and 67% (60–74) while patients at high risk by IPI had 3-year PFS and OS probabilities of 57% (50–64) and 68% (62–75). aTMTV attains the same prognostic information as manual TMTV on this dataset with 3-year PFS and OS of 54% and 64% (see details in the Supplemental Table 5). Using the proposed 10/30/60% stratification for high, intermediate and low risk groups proposed by the authors of IMPI [19], that patients in the high risk group by our proposed multivariate risk score including clinical and imaging metrics have lower 3 year PFS and 3 year OS (respectively 51.7% and 64.2%) than patients in the high risk group by IMPI (3-year PFS of 59.2% and 3-year OS of 70.5%, see details in Supplemental Table 6).

Previous studies have shown that high TMTV is associated with inferior survival outcomes in lymphoma [12, 32]. In the current study, we demonstrated that aTMTV was prognostic and correlated with known indicators of unfavorable survival outcomes such as LDH (Spearman *ρ* = 0.503; a correlation between LDH and extranodal involvement was also observed), as well as Ann Arbor stage and extranodal involvement (which was estimated by the combination of the tumor masses in the lungs, liver, spleen, and bone tumor involvement; we acknowledge that some physicians may consider spleen involvement as nodal involvement).

To assess the association between automatically extracted imaging information and CNS relapse, we analyzed a combined model of CNS-IPI and the model incorporating imaging and CNS variables to define three levels of risk. This combined model allowed us to identify a subpopulation of patients (5.5% of the total population) with a 2-year CNS-relapse probability of 17.1%. Compared with CNS-IPI alone, which separated the GOYA population into three risk-groups (2-year cumulative CNS relapse rates: 0.8%, 1.9% and 8.9%, respectively) [28], the combined model appeared to better identify patients at high risk of CNS relapse who may benefit from CNS prophylaxis and identifies a large subpopulation that could be spared from invasive diagnosis and CNS prophylaxis. These results show that fully automated assessments could further refine risk assessment for patients in a high-risk CNS-IPI group. Corroborating previous reports [33–, 34–36], we found that PET-CT identified more patients with bone marrow involvement than bone marrow biopsy, thus providing further evidence for the role of PET-CT in the staging of DLBCL. As shown in Fig. 1C, BMB and PET provide complementary yet overlapping information, patients that have both a positive biopsy and a positive PET have the worst prognosis, those with either a positive biopsy or a positive PET have an intermediate risk, and those with neither have the best outcome.

The analysis and interpretation of FDG-PET images in current clinical practice is performed by trained physicians and is mostly based on visual analysis and standardized uptake value assessment. Although it has not yet translated to clinical practice, when TMTV information is required, this is performed manually or using semi-automated analysis software [20], which is time-consuming and labor intensive; furthermore, the high degree of reproducibility that is of prime importance for these assessments is not always achievable with manual-based methods [20]. The fully automated segmentation algorithm described here has the potential to accurately assess tumor burden in patients with DLBCL, while providing substantial time savings and reduced work burden for physicians.

The algorithm used in this analysis was previously shown to have a high degree of correlation with conventionally measured TMTV performed by experienced nuclear medicine specialists [20]. In the present study, the prognostic utility of the algorithm was confirmed in patients with first-line DLBCL treated with R/G-CHOP; thus, AI-based algorithms were able to obtain fully automated TMTV measures in DLBCL that both correlate well with conventional measures of TMTV and provide relevant prognostic information.

Our findings generally agree with the literature for TMTV [12, 32]. The observation of a significant role of aTMTV in predicting survival outcomes in such a setting emphasizes the strength of aTMTV as a prognostic biomarker for identification of patients with DLBCL at high risk at the time of diagnosis, regardless of response to therapy [32]. Furthermore, the combination of TMTV with clinical variables has been shown to improve patient-risk stratification in a previous study of patients with peripheral T-cell lymphoma [37], which also supports the integration of automated imaging metrics into models comprised of clinical variables alone.

Limitations of this study included the multicollinearity among metrics (for example, IPI scores consider extranodal sites and LDH), which can undermine the statistical significance of an independent variable. Furthermore, there was a paucity of patients at a very high risk in the GOYA trial, as these patients typically require urgent treatment and therefore are not amenable to the delay to treatment initiation that is required for clinical trial enrollment. Other limitations include the need for a co-acquired CT scan along with the PET and the assessment of the proposed method only on clinical trial images following a standard imaging protocol. In addition, a minimal lesion size of 1 mL was used in the ground truth annotations; smaller lesions could be detected with more recent PET scanners.

We plan prospective validation of this fully automated FDG-PET/CT-based algorithm in external data sets (e.g. planned phase III DLBCL studies and data acquired as part of standard of care). Implementation into radiological and oncological workflows is needed to ensure maximal utility in clinical practice, and we are exploring making a research-only version of the software available on a limited basis to collaborating institutions.

## Conclusion

In this analysis of the GOYA trial, AI-enabled, fully automated baseline imaging metrics demonstrated prognostic value additional to standard clinical variables in predicting high-risk de novo DLBCL. These findings support the integration of both automated imaging and clinical factors in prognostic models to improve the accurate identification of patients with de novo DLBCL who are at high risk at a large scale, which could potentially assist in the development of optimized treatment strategies.

## Supplementary Information

**Additional file 1.**

## Data Availability

Researchers interested in testing and reviewing our models can request access to a separate work environment where they can utilize the models on a secure platform. Access can be requested for non-commercial/research use only purposes. Data sharing requests for patient-level Roche clinical trials data (excluding imaging) through a data request platform. At the time of writing this request platform is Vivli. https://vivli.org/ourmember/roche/. For up to date details on Roche's Global Policy on the Sharing of Clinical Information and how to request access to related clinical study documents, see here: https://go.roche.com/data_sharing. Requests for access to the imaging data should be directed to GLOFCT_PHC-data-sharing@msxdl.roche.com.
