# Supplementary material for: Full automation of total metabolic tumor volume from FDG-PET/CT in DLBCL for baseline risk assessments
Source: Cancer Imaging. 2022 Aug 12;22:39. doi: 10.1186/s40644-022-00476-0 (PMC9373298; doi:10.1186/s40644-022-00476-0)
Supplement: Supplementary file 1 — Additional file 1. [file 40644_2022_476_MOESM1_ESM.docx]

**Supplemental materials**

**Supplemental methods**

**Tumor segmentation algorithm**

The best performance for the Convolutional Neural Networks TMTV segmentation was obtained when Two-dimensional (2D) axial and sagittal slice-by-slice segmentations were averaged, followed by partition of the body into three different regions before refining the 2D predictions with region-specific three-dimensional (3D) convolutional neural networks. This process would allow for deep networks to overcome the highly unbalanced nature of the segmentation (small tumor to non-tumor volume in the whole body positron emission tomography [PET] scan) along with the limitations of current graphics processing unit memory capacity when dealing with large-size images. A multi-term loss and dilated convolutions^1^ allowed for the detection of small, localized and extensive disease-involvement tumors.^2^ We note that 2D coronal segmentations were not adding to the performance so were not included to maximize efficiency (minimizing processing time and resource consumption).

**Organ and location segmentation algorithm**

For automated organ and location segmentation, the lungs, liver, spleen, and bones were segmented using 3D UNets^3^ with four down-sampling and four up-sampling blocks, with two convolutional layers of 16 features per block, and a Leaky ReLU activation. The models were trained using the RMSProp optimizer and a Dice coefficient loss. The training dataset comprises 100 computed tomography scans from 100 patients in the GOYA training set^4,5^ and IMPower150 (NCT02366143)^6^ divided into 80 scans for training and 20 scans in the hold-out set. The Dice scores for the different organs are provided in Supplemental Table 1. In our previous work, this deep learning algorithm was shown to correlate with total metabolic tumor volume from the corresponding reported metrics in patients with follicular lymphoma.^2^

**Supplemental tables**

**Supplemental Table 1.** Dice Scores for organ/location segmentation

| **Organ/Location** | **Training set (*n* = 80)** | **Hold-out set (*n* = 20)** |
| --- | --- | --- |
| **Lungs** | 0.957 | 0.949 |
| **Bones** | 0.886 | 0.883 |
| **Spleen** | 0.924 | 0.915 |
| **Liver** | 0.938 | 0.931 |

**Supplemental Table 2.** HRs and 2-year PFS probability by extranodal involvement (fully automated assessment) in the full EP

| **Number of extranodal sites** | **HR (95% CI); *P*-value** | **2-year PFS probability, % (95% CI)** |
| --- | --- | --- |
| **0** | - | 78 (75-81) |
| **1** | 1.28 (1.02-1.61); .03 | 73 (69-78) |
| **2** | 1.68 (1.26-2.25); .0004 | 64 (56-72) |
| **3+** | 2.15 (1.47-3.15); < .0001 | 62 (51-76) |

CI, confidence interval; EP, evaluable population; HR, hazard ratio; PFS, progression-free survival.

**Supplemental Table 3.** 2-year PFS probability by BMB and bone involvement (fully automated assessment) in the full EP

| **BMB and bone involvement** | ***n* (%)** | **2-year PFS probability, % (95% CI)** |
| --- | --- | --- |
| **BMB (-) and bone involvement (-)** | 887 (70.0) | 77 (74-80) |
| **BMB (-) and bone involvement (+)** | 224 (17.7) | 69 (64-76) |
| **BMB (+) and bone involvement (-)** | 86 (6.8) | 69 (60-80) |
| **BMB (+) and bone involvement (+)** | 71 (5.6) | 62 (51-75) |

BMB, bone marrow biopsy; CI, confidence interval; EP, evaluable population; PFS, progression-free survival.

**Supplemental Table 4.** Comparison of prognosis of manual and automated TMTV on the BEP (N=1,268)

|  | **PFS HR (95% CI)** | **OS HR (95% CI)** |
| --- | --- | --- |
| **Manual TMTV**  Median Split  Third quartile Split | 1.76 (1.42-2.17)  1.96 (1.60-2.41) | 1.83 (1.38-2.39)  2.11 (1.64-2.72) |
| **Automated TMTV**  Median Split  Third quartile Split | 1.70 (1.39-2.07)  1.94 (1.58-2.38) | 1.72 (1.34-2.21)  2.03 (1.58-2.62) |

**Supplemental Table 5.** Comparison of prognosis of the proposed International Metabolic Prognostic Index (IMPI) using manual and auto TMTV with IPI on the the BEP (N=1,268) using the same group sizes as IPI

|  | **PFS** | | | **OS** | |
| --- | --- | --- | --- | --- | --- |
|  | ***N*** | **HR (95% CI)** | **3-year PFS,  % (95% CI)** | ***n*** | **3-year OS,  % (95% CI)** |
| **IMPI (manual)** |  |  |  |  |  |
| Low | 253 | 0.34 (.24-.48) | 83 (78-88) | 253 | 90 (86-94) |
| Low-Int | 456 | 0.51 (.39-.66) | 74 (70-78) | 456 | 84 (80-87) |
| High-Int | 369 | .71 (.54-.93) | 66 (61-71) | 369 | 82 (78-86) |
| High | 190 | Reference | 55 (48-62) | 190 | 67 (60-74) |
| **IMPI (auto)** |  |  |  |  |  |
| Low | 253 | 0.32 (.23-.45) | 83 (79-89) | 253 | 90 (86-94) |
| Low-Int | 456 | 0.44 (.34-.59) | 75 (71-79) | 456 | 84 (81-88) |
| High-Int | 369 | .71 (.54-.92) | 65 (60-70) | 369 | 82 (78-86) |
| High | 190 | Reference | 54 (47-61) | 190 | 65 (58-72) |
| **IPI** |  |  |  |  |  |
| Low | 253 | 0.28 (.19-.40) | 85 (80-89) | 253 | 93 (90-97) |
| Low-Int | 456 | 0.58 (.34-.59) | 72 (67-76) | 456 | 83 (80-87) |
| High-Int | 369 | .69 (.52-.90) | 67 (62-72) | 369 | 80 (76-84) |
| High | 190 | Reference | 57 (50-64) | 190 | 68 (62-75) |

**Supplemental Table 6.** Comparison of prognosis of the proposed International Metabolic Prognostic Index (IMPI) using manual and our multivariate risk score including imaging metrics and clinical variables on the the test set (N=422) using the same group sizes as IMPI

|  | **PFS** | | | **OS** | |
| --- | --- | --- | --- | --- | --- |
|  | ***N*** | **HR (95% CI)** | **3-year PFS,  % (95% CI)** | ***n*** | **3-year OS,  % (95% CI)** |
| **IMPI (manual)** |  |  |  |  |  |
| Low | 253 | 0.55 (.33-.93) | 77 (72-83) | 253 | 85 (81-90) |
| Int | 126 | .80 (.46-1.39) | 68 (60-77) | 126 | 81 (75-89) |
| High | 43 | Reference | 59 (46-77) | 43 | 71 (58-86) |
| **Ours** |  |  |  |  |  |
| Low | 261 | 0.36 (.21-.59) | 80 (75-85) | 261 | 87 (84-92) |
| Int | 120 | .72 (.43-1.21) | 64 (55-73) | 120 | 78 (71-86) |
| High | 41 | Reference | 52 (38-71) | 41 | 65 (51-81) |

**Supplemental figures**
**Supplemental Figure 1.** KM curves comparing PFS in the GOYA study for the EP with (A) the SEP and with (B) the non-EP.

| **A** |
| --- |
| 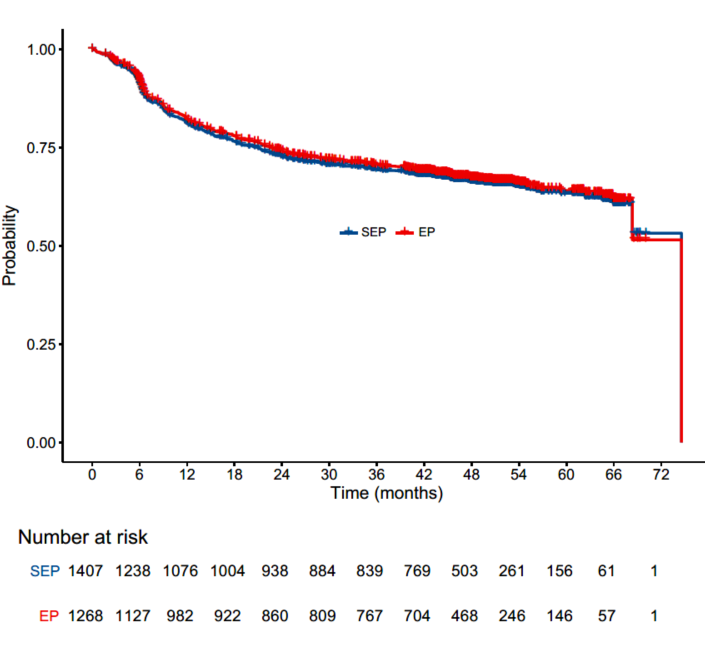 |
| **B** |
| 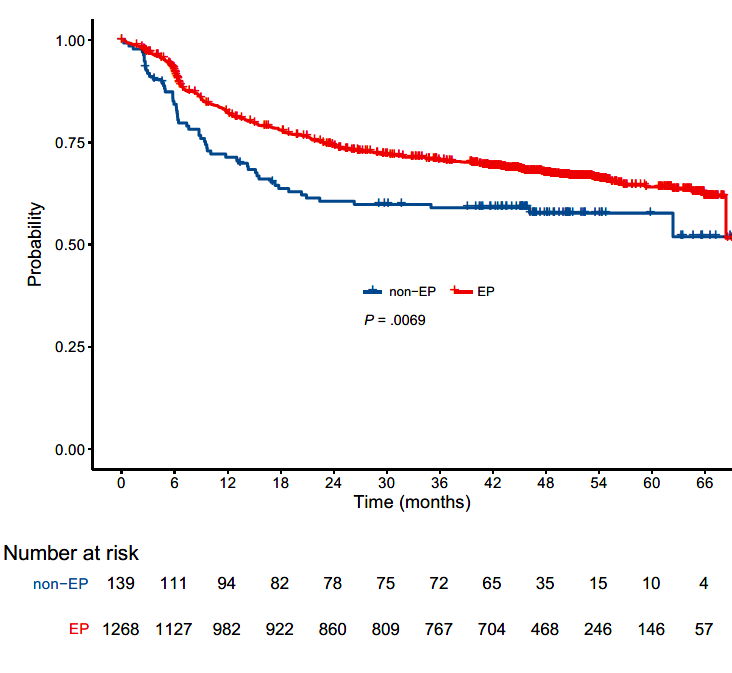 |

EP, evaluable population; KM, Kaplan–Meier; PFS, progression-free survival; SEP, safety-evaluable population.

**Supplemental Figure 2.** Box plot of aTMTV by number of extranodal sites involved.

| 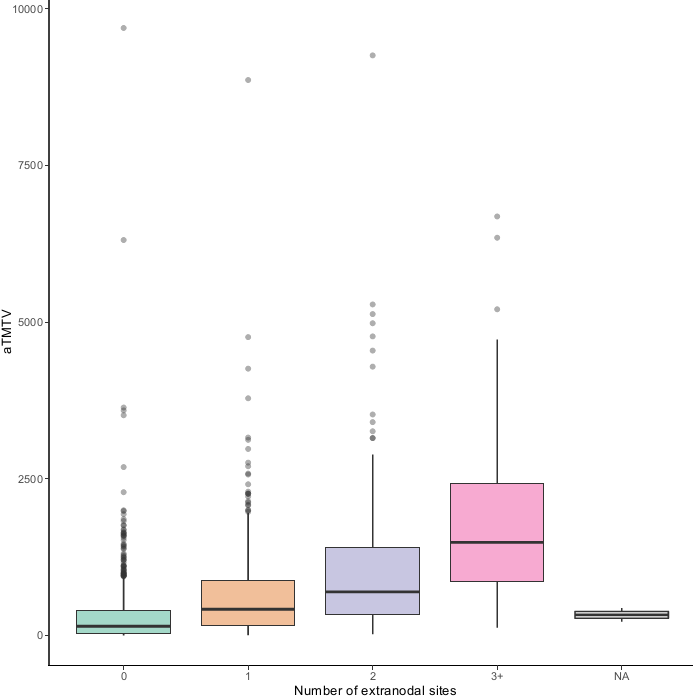 |
| --- |

aTMTV, automated total metabolic tumor volume; NA, not available.

**Supplemental Figure 3.** (A) Relationship between LDH and aTMTV in the full EP. (B) Boxplot of log LDH (left) and LDH (right) by number of extranodal sites*.

| **A** |
| --- |
| **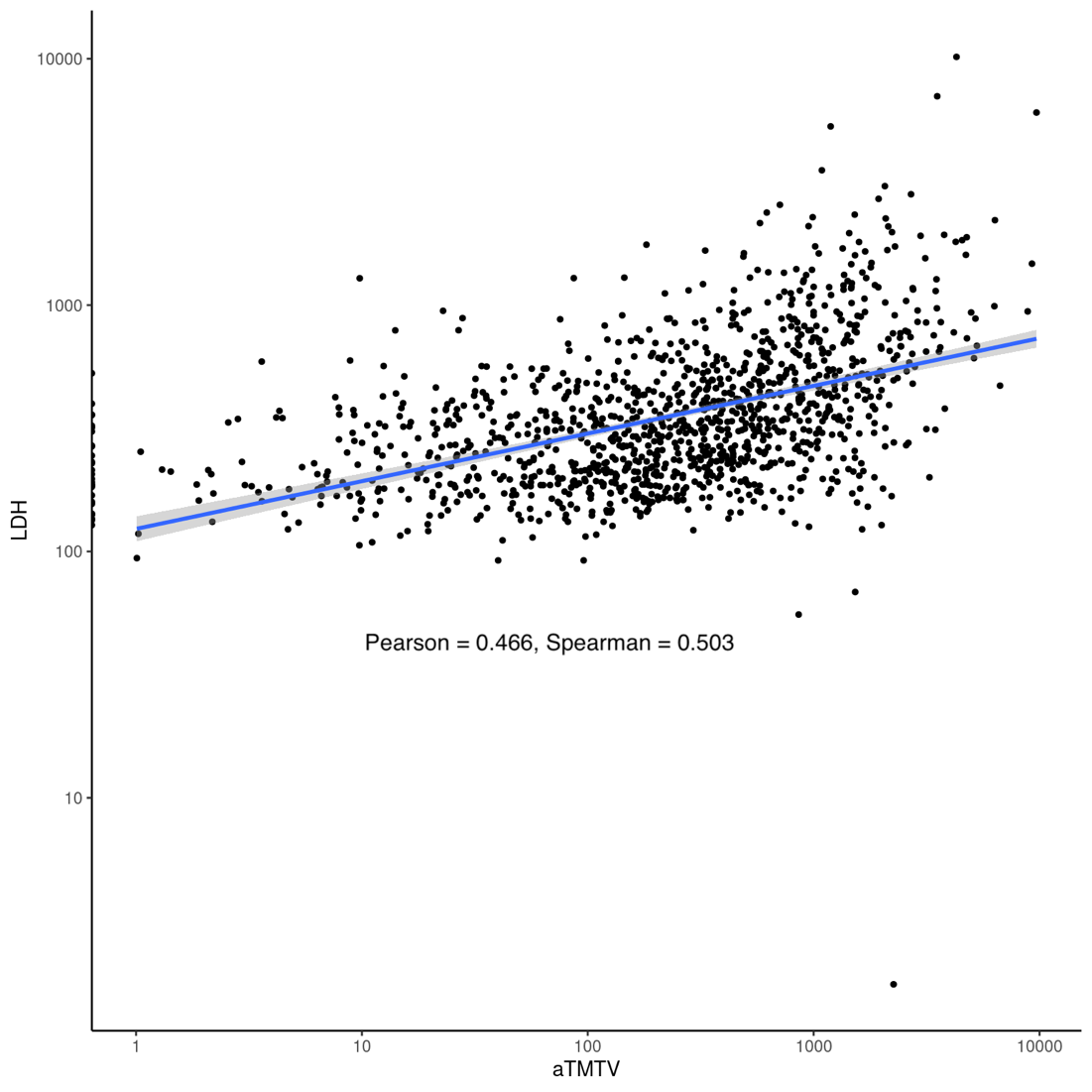** |
| **B** |
| 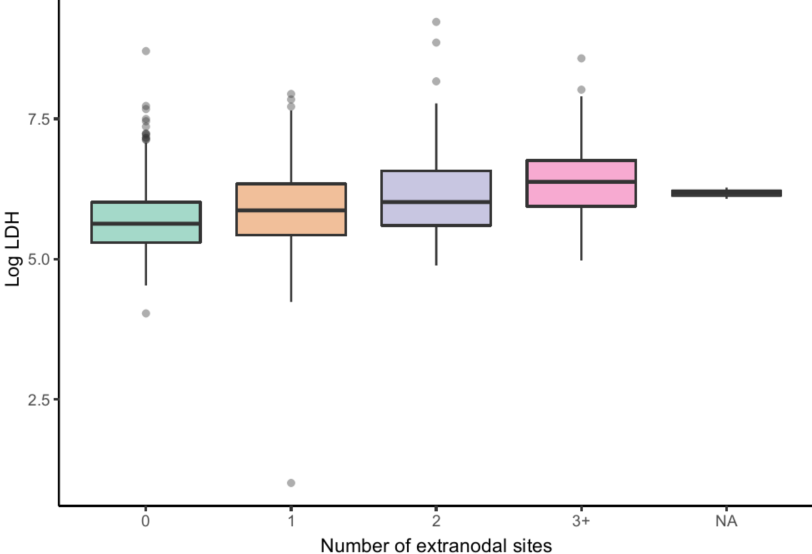 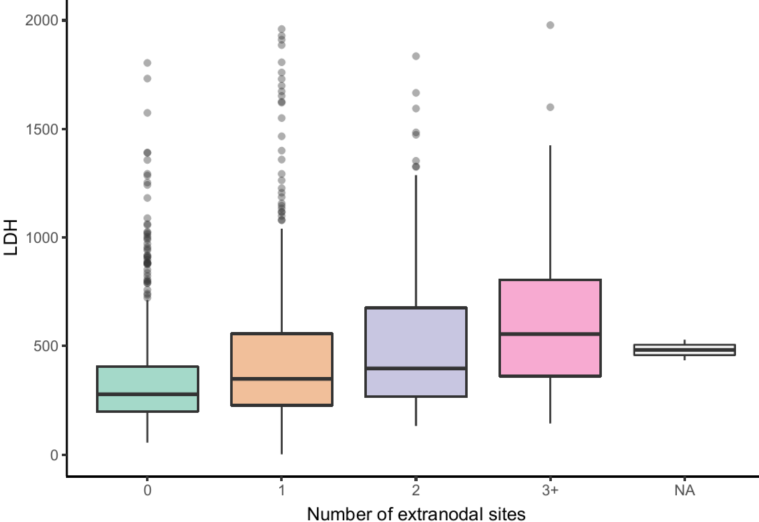 |

*Extranodal involvement was estimated by the combination of the tumor masses in the lungs, liver, spleen and bone tumor involvement.

EP, evaluable population; LDH, lactate dehydrogenase; aTMTV, automated total tumor metabolic volume.

**Supplemental Figure 4.** Boxplot of the association between aTMTV (mL) and Ann Arbor stage.

| 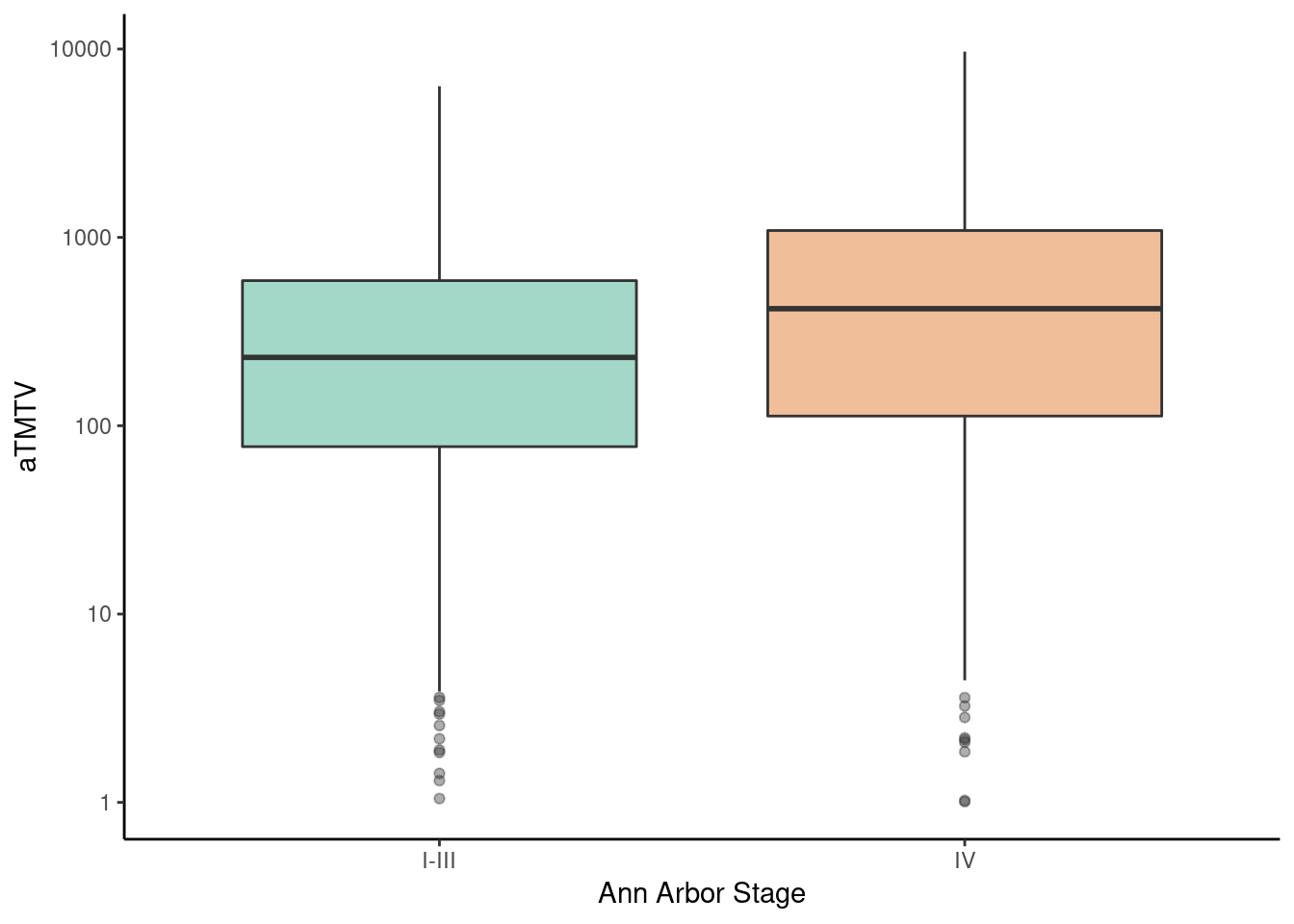 |
| --- |

aTMTV, automated total metabolic tumor volume.

**Supplemental Figure 5.** (A) Model selection with LASSO regression and (B) multivariate Cox PH model with LASSO penalty*.

| **A** |
| --- |
| 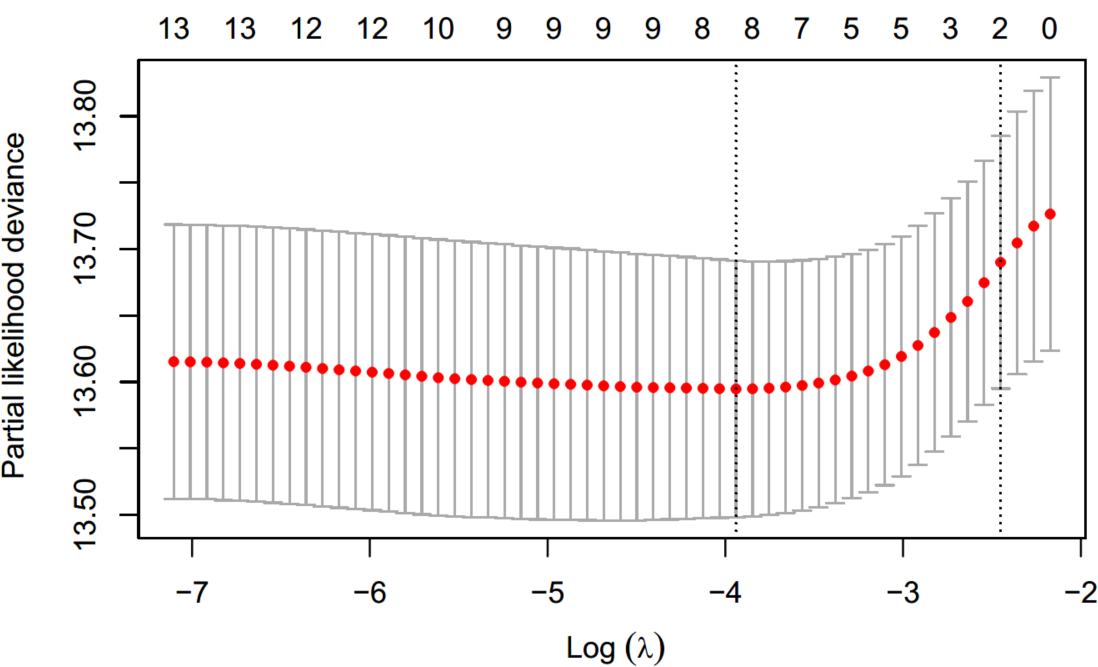 |
| **B** |
| 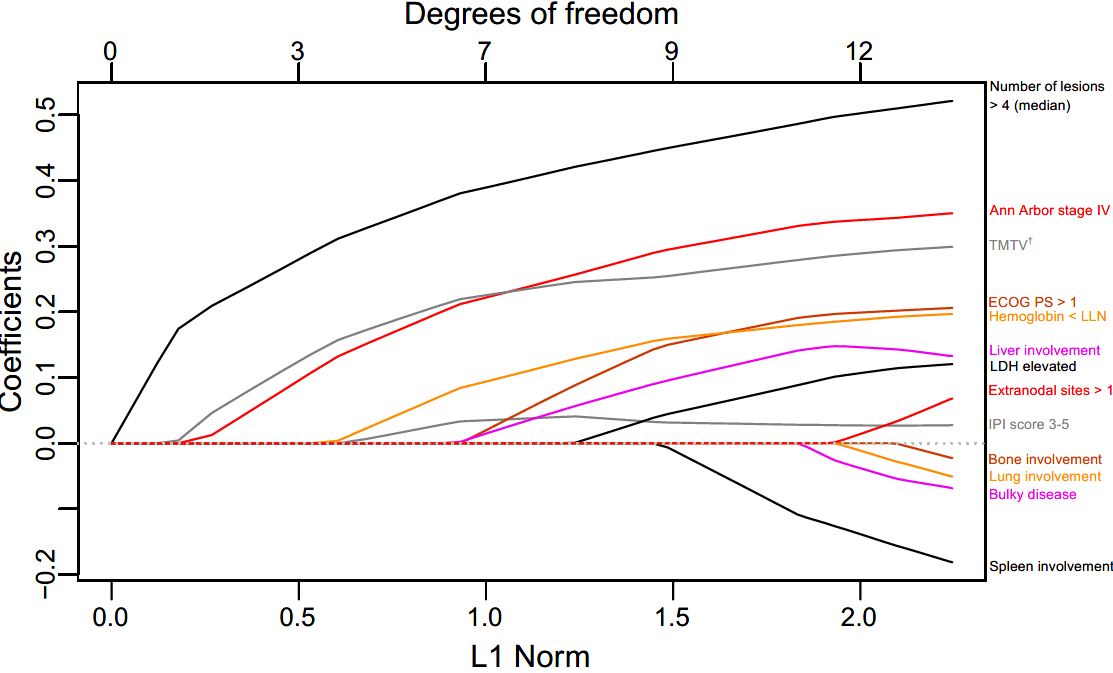 |

*Using Cox partial likelihood; ^†^Thresholds of both median and Q3 were considered for TMTV.

ECOG PS, Eastern Cooperative Oncology Group performance status; IPI, International Prognostic Index; LDH, lactate dehydrogenase; LLN, lower limit of normal; PH, proportional hazard; Q3, third quartile; TMTV, total metabolic tumor volume.

**Supplemental Figure 6.** ROC for investigator-assessed PFS in the test set for the model including imaging metrics and the model including clinical variables only.

|  |
| --- |
| 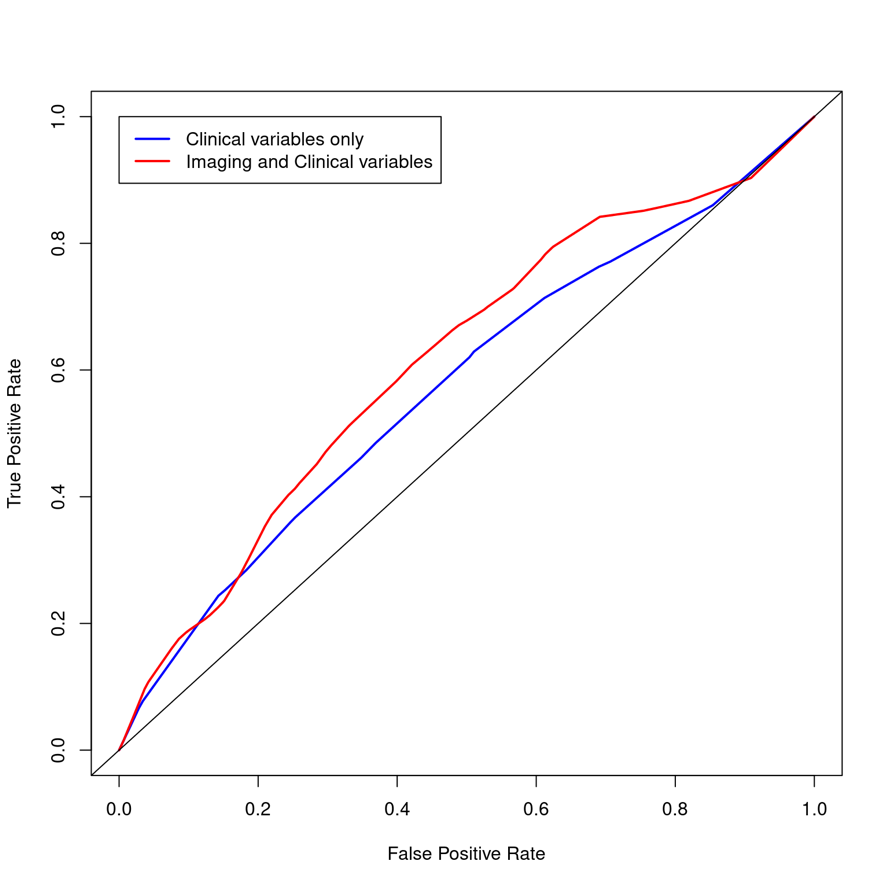 |
|  |
|  |

PFS, progression-free survival; ROC, receiver operating characteristics.

**References**

1. Yu F, V. K. Multi-scale context aggregation by dilated convolutions. ICLR 2016. Available at: https://arxiv.org/abs/1511.07122. Accessed March 2021.

2. Jemaa S, Fredrickson J, Carano RAD, Nielsen T, de Crespigny A, Bengtsson T. Tumor segmentation and feature extraction from whole-body FDG-PET/CT using cascaded 2D and 3D convolutional neural networks. J Digit Imaging. 2020; 33(4): 888-894.

3. Ronneberger O, Fischer P, Brox T. U-Net: convolutional networks for biomedical image segmentation. 2015. Available at: https://arxiv.org/abs/1505.04597. Accessed February 2021.

4. Vitolo U, Trneny M, Belada D et al. Obinutuzumab or rituximab plus cyclophosphamide, doxorubicin, vincristine, and prednisone in previously untreated diffuse large B-cell lymphoma. J Clin Oncol. 2017; 35(31): 3529-3537.

5. Sehn LH, Martelli M, Trněný M et al. A randomized, open-label, Phase III study of obinutuzumab or rituximab plus CHOP in patients with previously untreated diffuse large B-Cell lymphoma: final analysis of GOYA. J Hematol Oncol. 2020; 13(1): 71.

6. Socinski MA, Jotte RM, Cappuzzo F et al. Atezolizumab for first-line treatment of metastatic nonsquamous NSCLC. N Engl J Med. 2018; 378(24): 2288-2301.
